# Associations Between Emotion Regulation and Life Satisfaction Among University Students From Germany, Hong Kong, and Japan: The Mediating Role of Social Support

**DOI:** 10.3389/fpsyg.2021.745888

**Published:** 2021-10-18

**Authors:** Fabian Schunk, Gisela Trommsdorff, Natalie Wong, Gen Nakao

**Affiliations:** ^1^Department of Psychology, University of Konstanz, Konstanz, Germany; ^2^Department of Psychology, The Chinese University of Hong Kong, Hong Kong, China; ^3^Department of Management, Otemon Gakuin University, Ibaraki, Japan

**Keywords:** emotion regulation, suppression, life satisfaction, perceived social support, culture

## Abstract

The social adaptiveness of emotion regulation (ER) may differ across cultures due to different social values for the experience and expression of emotions. Specifically, suppression might disrupt social interactions among Germans, but not among Hong Kong Chinese (HKC) and Japanese, due to an emphasis on self-expression and authenticity in Western cultures. In the present study, we examined cultural differences in associations of ER strategies with life satisfaction and social support. Extending prior research, we also test whether social support functions as a mediator for relationships between ER strategies and life satisfaction within cultural groups. University students from Germany (*N* = 148), Hong Kong (*N* = 125), and Japan (*N* = 127) participated in our online survey. Moderation analyses revealed that suppression was related to lower life satisfaction and less social support among Germans, but not among HKC nor Japanese. Social support completely mediated the negative relationship between suppression and life satisfaction among Germans. Furthermore, for Germans and HKC, social support partially mediated the positive relationship between reappraisal and life satisfaction, and the negative relationship between rumination and life satisfaction. Our findings suggest that cultural differences in the associations between ER and well-being might be largely explained by the differential effect of ER strategies on social functioning and adaptation in the respective cultural context.

## Introduction

Geteiltes Leid ist halbes Leid [A sorrow shared is a sorrow halved]

—German proverb

勝人者有力, 自勝者強 [He who controls others may be powerful, but he who has mastered himself is mightier still]

—Lao Tzu, Chinese philosopher

顔で笑って心で泣いて [Smiling face, crying heart]

—Japanese proverb

Emotion regulation (ER) describes “processes by which individuals influence which emotions they have, when they have them, and how they experience and express these emotions” (Gross, 1998, p. 275). The usage of specific ER strategies and their associations with life satisfaction has been found to differ across cultures (see Ford and Mauss, 2015, for a review). One ER strategy that gained particular attention in recent years is *suppression* (i.e., inhibiting the expression of emotions; Gross and John, 2003). In Western cultures, suppression is often considered maladaptive due to its negative association with well-being among Western participants (see Joormann and Stanton, 2016, for a review). However, negative relations between suppression, and well-being were found to be absent among participants from Hong Kong (Soto et al., 2011), Japan (Yoshizu et al., 2013), and Singapore (Su et al., 2013). Yet, much about the underlying mechanisms of these cultural differences remains to be understood.

Past research suggests that the cultural fit of emotions, defined as “the compatibility between a person's emotions and cultural norms and values” (Yoo and Miyamoto, 2018, p. 2), promotes well-being by shaping emotional experiences in culturally valued ways (De Leersnyder et al., 2013; Mesquita et al., 2016; Yoo and Miyamoto, 2018). Since ER often occurs in social interactions (English and Eldesouky, 2020), cultural differences in the social adaptiveness of a given ER strategy may explain the different effects of ER on well-being across cultures. Specifically, if a selected ER strategy is consistent with one's cultural values, it could promote the attainment of relational resources. As such, using certain ER strategies may result in social benefits (e.g., social support) which may then positively influence an individual's well-being. Examining the potential mediating role of social functioning in the relationship between ER and well-being in various cultures might be the key to understand the socio-cultural functions of ER.

According to many cultural psychologists, Western European cultures emphasize self-expression, authenticity, individuality, and self-enhancement, whereas East Asian cultures value self-restraint, social harmony, interdependence, and self-effacement (Markus and Kitayama, 1991; Trommsdorff and Rothbaum, 2008). Suppression may interfere with social functioning in Western cultures by disrupting the socially valued authentic expressions of emotions, making individuals appear distant and unfaithful to their true selves (English et al., 2012). Concurrent empirical evidence from US-Americans suggests that individuals who frequently suppress their emotions are less likely to share negative and positive emotions and report higher attachment avoidance (Gross and John, 2003). Masking emotional expressions during interactions has been shown to disrupt communication and increase blood pressure in the suppressor and the interaction partner (Butler et al., 2003). In Western cultures, individuals might expect others to share their emotions to create a personal connection. The above-stated German proverb (“A sorrow shared is a sorrow halved”) illustrates that sharing even negative emotions may induce benefits in this cultural context. This may apply to the German culture in particular, as Germans were shown to avoid negative emotions less than US-Americans (Koopmann-Holm and Tsai, 2014).

In contrast, in East Asian cultures that value interdependence and social connectedness, suppressing negative and potentially disruptive emotions might be socially promoted to maintain harmony (Trommsdorff and Rothbaum, 2008; Friedlmeier et al., 2011). Previous research found that individuals from Hong Kong (Soto et al., 2011) and Japan (Matsumoto, 2006) are more likely to suppress their emotions compared to US-Americans. Suppression might be normative in East Asian (vs. Western) cultural contexts since concerns about burdening others are crucial for functioning in interdependent cultural contexts in which well-being is largely based on relational harmony (Hitokoto and Uchida, 2018). Accordingly, East Asian interaction partners might be more accepting of emotional suppressive communication styles. In line with this reasoning, Butler et al. (2007) demonstrated differences in the social consequences of suppression in a diverse sample of US-American women. Suppression among participants holding European values resulted in less responsiveness during face-to-face interaction and in more negative and hostile perceptions by the partner. These negative effects were reduced among participants with more Asian values. In another more recent study, suppression was related to low conflict resolution after a disagreement among Belgian couples, whereas no effect was observed among Japanese couples (Schouten et al., 2020).

De Leersnyder et al. (2013) emphasize that cultural differences in ER can already occur during emotion elicitation. *Rumination* (i.e., repeatedly focusing attention on causes and consequences of negative emotions) and *reappraisal* (i.e., modifying the meaning of an emotional situation) describe two antecedent-focused ER strategies that target processes before emotional responses (McRae and Gross, 2020). While both appear to have similar relations with well-being across cultures, with rumination being related to lower (Chang et al., 2010) and reappraisal to higher well-being (Kwon et al., 2013), their associations with social support and the strengths of these relations may differ. For instance, Chang et al. (2010) found a stronger negative link between rumination and life satisfaction among European Americans compared to Asian Americans. Western studies suggest that individuals who ruminate lose social support as they behave in socially obtrusive ways (e.g., have clingy and aggressive tendencies; Nolen-Hoeksema et al., 2008). In East Asian cultures, these negative effects might be reduced due to values emphasizing self-effacement and self-criticism (Heine et al., 1999; Chang et al., 2010). Ruminating through focusing on one's shortcomings may even serve some social functions by adjusting to other's expectations (De Leersnyder et al., 2013), which may buffer negative social consequences of rumination in interdependent cultures. In contrast, positive associations between reappraisal and social support among US-American (Gross and John, 2003) and Japanese samples (Urano and Ikeda, 2020) suggest a universal social function of reappraisal. Reappraisal is effective for reducing negative emotions and influencing emotional behavior (for a meta-analysis, see Webb et al., 2012). Habitual reappraisers may thus appear more friendly and pleasant to interact with. Since social support is known to be an important predictor of well-being (Cohen and Wills, 1985), the social conditions of ER may at least partially explain how ER strategies relate to well-being.

In sum, specific ER strategies might be similarly or differently related to well-being across cultures through the mediating effect of social support. Social support is conceptualized here as social functioning, whereas life satisfaction will be used as an indicator for well-being (see Diener et al., 2018, for a review on the conceptualization of well-being). We will examine the mediating role of social support across cultures by comparing individuals from Germany, Hong Kong, and Japan to increase cultural variance. Germany represents a Western and independent cultural context that values autonomy and harmony and is moderately high on survival/self-expression values (Schwartz, 2006; Friedlmeier et al., 2008). In turn, both Hong Kong and Japan represent East Asian and interdependent cultural contexts with a particular emphasis on embeddedness. Notably, Japanese culture places more value on harmony and intellectual autonomy, and less value on embeddedness and hierarchy, compared to Hong Kong (Schwartz, 2006). Hong Kong and Japan also differ historically. Specifically, living under British sovereignty for many years might have promoted different values in Hong Kong compared to other East Asian cultures (Kobayashi et al., 2021). By including two East Asian cultural contexts, we can empirically test whether ER functions differently within this region, thereby going beyond commonly used East-West dichotomies (see Vignoles et al., 2016). We further aim to extend previous findings by focusing on Germany as a Western culture, instead of the frequently examined USA.

In line with the assumption that suppression disrupts social functioning in Western European (but not East Asian) cultures, we predicted: suppression will be related to lower life satisfaction through less social support among Germans, but not among Hong Kong Chinese (HKC) or Japanese (hypothesis 1). Consistent with previous research linking reappraisal to higher and rumination to lower well-being across cultures, we further predicted: reappraisal will be related to higher life satisfaction through more social support across cultures (hypothesis 2), and rumination will be related to lower life satisfaction through less social support across cultures (hypothesis 3). We will further explore whether culture moderates the strengths of the predicted relationships. For instance, rumination may have a weaker effect on life satisfaction in East Asian (vs. Western) cultures through the lower mediating effect of social support. This study was preregistered on the Open Science Framework before data collection (https://osf.io/ks6jx/; see Supplementary Material for comments and additional analyses).

## Method

### Participants

Our final sample consisted of 400 university students (*M*_*age*_ = 20.71 years, *SD* = 2.42; 60.3% female): Participants were 148 Germans (*M*_*age*_ = 22.72 years, *SD* = 2.28; 70.3% female), 125 HKC (*M*_*age*_ = 20.38 years, *SD* = 1.55; 68.8% female), and 127 Japanese (*M*_*age*_ = 18.69 years, *SD* = 1.07; 40.2% female, 0.8% diverse). Participants were included when they replied to at least 80% of all items and were not younger than 18 years and not older than 29 years. One Japanese participant was excluded since he answered every item the same. As outlined in our preregistration, we aimed for a sample size of 120 participants per culture based on an a priori power analysis. The study was approved by the Ethics Committee of the University of Konstanz.

### Procedure

We collected data from July to November 2020 using the online platform SoSci Survey (https://www.soscisurvey.de/). Participants gave their informed consent and voluntarily participated in the study. Compensation differed across cultures to match regional circumstances: Japanese received course credits, whereas HKC could win a voucher for an online marketplace. Germans received either course credits or a chance to win an Amazon voucher of similar value. German university students were recruited in Constance, Cologne, and Heidelberg. Additionally, the study was advertised in Germany on the website SurveyCircle (https://www.surveycircle.com/de/). We recruited HKC through mass mail at the Chinese University of Hong Kong. Japanese participants were recruited at Otemon Gakuin University and Kwansei Gakuin University.

### Measures

Participants completed the scales in German, Chinese, or Japanese, respectively. We either applied existing language versions or translated (and back-translated) scales when necessary. Further information on the scales, including sample items and applied language versions, are given in the Supplementary Material. We made each survey available online before data collection (https://osf.io/ks6jx). Cronbach's alphas are presented in Table 1.

**Table 1:** Descriptive statistics and congruence coefficients for study variables by culture.

**Variables**	**German (*****n*** **=** **148)**			**Hong Kong (*****n*** **=** **125)**			**Japanese (*****n*** **=** **127)**		
	***M*** **(*SD*)**	**α**	**ϕ**	***M*** **(*SD*)**	**α**	**ϕ**	***M*** **(*SD*)**	**α**	**ϕ**
Suppression	3.53 (1.12)	0.73	0.96	3.85 (1.11)	0.75	0.98	3.70 (1.18)	0.71	0.98
Reappraisal	4.68 (1.08)	0.82	0.98	4.65 (0.86)	0.83	0.98	4.43 (1.06)	0.78	0.99
Rumination	3.35 (0.73)	0.79	1	3.28 (0.72)	0.83	1	3.28 (0.90)	0.88	1
Social support	5.70 (0.99)	0.85	1	4.84 (1.10)	0.89	1	5.73 (1.05)	0.90	1
Life satisfaction	5.24 (1.08)	0.85	0.99	3.99 (1.13)	0.84	1	3.99 (1.27)	0.84	1

*α, Cronbach's alpha; ϕ, Congruence coefficient (Tucker's phi) rotated to the average factor solution across cultures*.

#### Emotion Regulation

Participants completed the Emotion Regulation Questionnaire (ERQ; Gross and John, 2003) to measure habitual use of suppression (4 items) and reappraisal (6 items; 1 = *strongly disagree*, 7 = *strongly agree*). The ERQ is a well-established measure of trait ER that has been used across various cultures. This allows interpreting our findings in the context of previous cross-cultural studies (e.g., Soto et al., 2011; Kwon et al., 2013). Five items from the Perseverative Thinking Questionnaire (PTQ; Ehring et al., 2011) were used to measure rumination (0 = *never*, 4 = *almost always*).

#### Perceived Social Support

Participants completed eight items from the Multidimensional Scale of Perceived Social Support (MSPSS; Zimet et al., 1988) to measure perceived social support (1 = *strongly disagree*, 7 = *strongly agree*). The MSPSS provides a total score for social support by averaging perceived social support from the sources family, friends, and significant others. However, we only used items referring to family and friends due to concerns about the comparability of items related to significant others (e.g., participants might define significant others differently across cultures).

#### Life Satisfaction

The 5-item Satisfaction With Life Scale (SWLS; Diener et al., 1985) was used to measure a participant's judgment of satisfaction with their life (1 = *strongly disagree*, 7 = *strongly agree*).

### Data Processing and Analytic Strategies

When participants skipped a page without replying to every item, the online survey displayed a message reminder. Consequently, only two missing values emerged for the whole dataset. Missing values were estimated using expectation maximization. We conducted analyses with IBM SPSS Statistics version 27 and used R version 3.6.3 for measurement equivalence testing. The SPSS macro PROCESS version 3.5 (Hayes, 2017) was used to run moderation (PROCESS model 1) and mediation analyses (PROCESS model 4). We included age and gender as covariates in all analyses. Culture was dummy-coded (1 = *Germany*, 2 = *Hong Kong*, 3 = *Japan*).

### Cultural Measurement Equivalence

To examine structural measurement equivalence across cultures, we calculated congruence coefficients (Tucker's phi) for each instrument following the procedure described by Fischer and Karl (2019). We created a pan-cultural correlation matrix that weighted each cultural group equally and used the resulting average matrix within our principal factor analysis as a reference for the individual sample's factor structures. The advantage of this approach is that no cultural group is prioritized. Congruence coefficients and descriptive statistics are displayed in Table 1. All scales demonstrated structural equivalence across cultures as each congruence coefficient was above 0.95 (van de Vijver and Leung, 1997).

## Results

First, we conducted moderation analyses for each ER strategy predicting life satisfaction and social support, respectively, to examine whether life satisfaction and social support are differently related to ER across cultures. In cases of significant relationships, we ran mediation analyses (bootstrapping with 5,000 resamples) to test whether social support mediates the respective relationships within cultural groups. In addition, partial correlations (controlled for age and gender) among variables are presented in the Supplementary Material. Notably, the positive link between social support and life satisfaction was not moderated by culture (*p* = 0.416).

### Suppression

**Figure 1:**
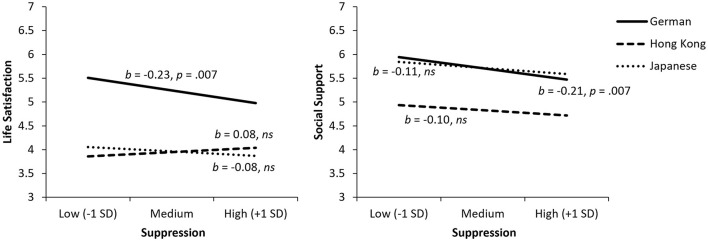
Relationships of suppression with life satisfaction (left) and social support (right). ns, not statistically significant.

Culture moderated the relationship between suppression and life satisfaction, *F*(2, 392) = 3.06, *p* = 0.048, ΔR^2^ = 0.012 (see Figure 1). We decomposed the interaction by examining the conditional effects of suppression in each cultural group. Suppression was related to lower life satisfaction among Germans (*b* = −0.23, *p* = 0.007), but unrelated to life satisfaction among HKC (*p* = 0.404) and Japanese (*p* = 0.361). We observed a similar pattern for social support as an outcome. Although the omnibus interaction test failed to reach significance (*p* = 0.540), the conditional effects revealed a significant and negative relationship between suppression and social support for Germans (*b* = −0.21, *p* = 0.007) and no effects for HKC (*p* = 0.255) and Japanese (*p* = 0.155). A mediation analysis further revealed a significant indirect effect of suppression on life satisfaction through social support in the German sample, *b* = −0.11, 95% CI [−0.19, −0.03]. The direct effect of suppression became non-significant, indicating a complete mediation. Hypothesis 1 was therefore supported.

### Reappraisal

**Figure 2:**
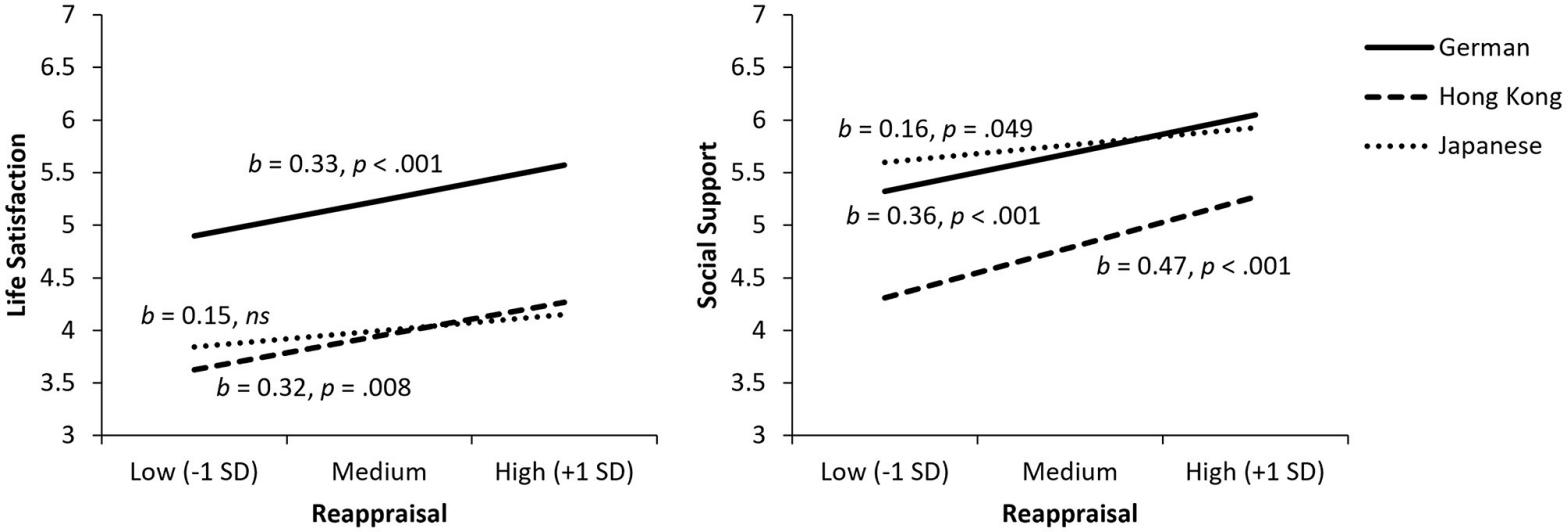
Relationships of reappraisal with life satisfaction (left) and social support (right). ns, not statistically significant.

Culture did not moderate the link between reappraisal and life satisfaction (*p* = 0.326). Yet, the conditional effects showed that reappraisal was related to higher life satisfaction among Germans (*b* = 0.33, *p* < 0.001) and HKC (*b* = 0.32, *p* = 0.008), but not among Japanese (*p* = 0.110; see Figure 2). Culture moderated the relationship between reappraisal and social support, *F*(2, 392) = 3.04, *p* = 0.049, ΔR^2^ = 0.012, indicating a significantly weaker effect among Japanese compared to HKC (Germans: *b* = 0.36, *p* < 0.001; HKC: *b* = 0.47, *p* < 0.001; Japanese: *b* = 0.16, *p* = 0.049). Because reappraisal was unrelated to life satisfaction among Japanese, we tested mediation effects only for Germans and HKC. For both groups, social support completely mediated the positive link between reappraisal and life satisfaction, *b* = 0.17, 95% CI [0.09, 0.28] for Germans; *b* = 0.26, 95% CI [0.14, 0.42] for HKC. Thus, hypothesis 2 was supported for Germans and HKC, but not for Japanese.

### Rumination

**Figure 3:**
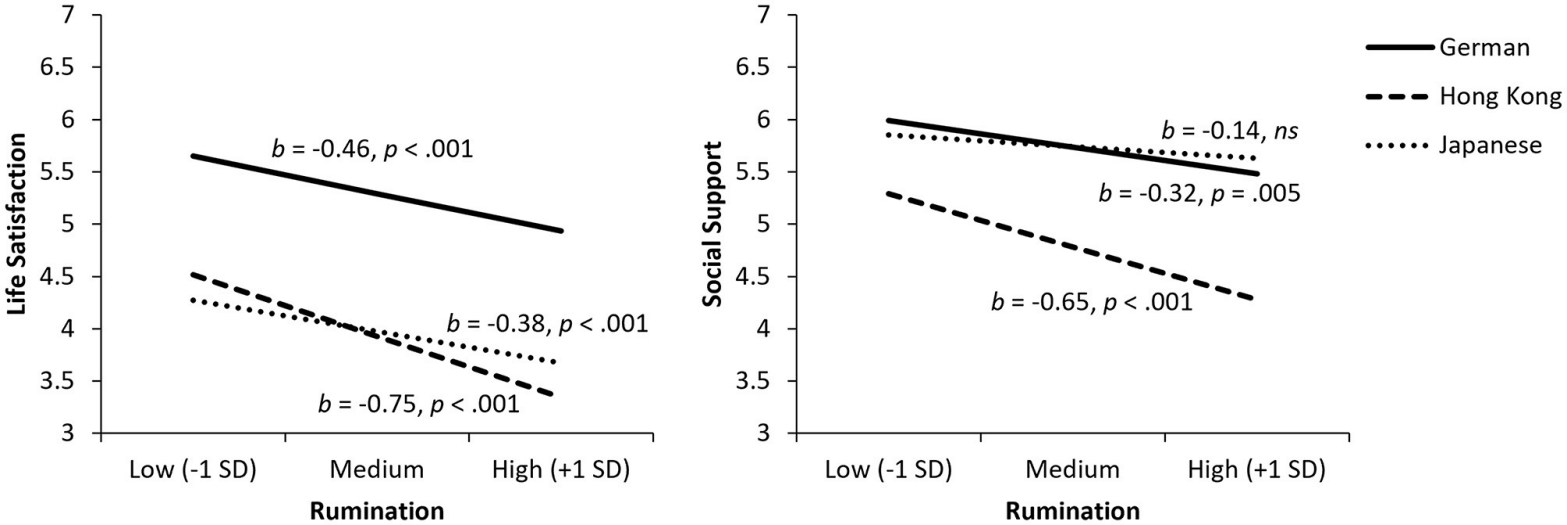
Relationships of rumination with life satisfaction (left) and social support (right). ns, not statistically significant.

Culture did not moderate the link between rumination and life satisfaction (*p* = 0.098). Rumination was associated with lower life satisfaction across cultural groups (Germans: *b* = −0.46, *p* < 0.001; HKC: *b* = −0.75, *p* < 0.001; Japanese: *b* = −0.38, *p* < 0.001; see Figure 3). For social support, we obtained a significant cultural moderation, *F*(2, 392) = 5.01, *p* = 0.007, ΔR^2^ = 0.020. Rumination was related to less social support among Germans (*b* = −0.32, *p* = 0.005) and HKC (*b* = −0.65, *p* < 0.001), but unrelated among Japanese (*p* = 0.147). We tested mediation effects only for Germans and HKC as rumination was unrelated to social support among Japanese. Social support partially mediated the negative link between rumination and life satisfaction among Germans, *b* = −0.16, 95% CI [−0.31, −0.06], and HKC, *b* = −0.30, 95% CI [−0.47, −0.13]. Therefore, hypothesis 3 was supported for Germans and HKC, but not for Japanese.

## Discussion

In the present study, we examined associations between ER, life satisfaction, and perceived social support among university students from Germany, Hong Kong, and Japan. Our findings extend previous research by testing social support as a mediator for relationships between distinct ER strategies and life satisfaction across cultural groups. As predicted, suppression was related to lower life satisfaction through less social support among Germans, but not among Hong Kong Chinese (HKC) or Japanese (hypothesis 1). Furthermore, among Germans and HKC, reappraisal was positively related to life satisfaction through more social support, whereas rumination was related to lower life satisfaction through less social support. For Japanese, reappraisal was associated with more social support, but unrelated to life satisfaction. Similarly, for Japanese, rumination was associated with lower life satisfaction, but unrelated to social support. Thus, hypotheses 2 and 3 were only partially supported.

Suppression has been frequently linked to lower well-being and impaired social functioning in Western, but not in East Asian cultures (Soto et al., 2011; Su et al., 2013; Yoshizu et al., 2013). We replicated this pattern by linking suppression to lower life satisfaction and less social support among Germans, but not among Japanese or HKC. Extending previous studies, we found that social support completely mediated the negative link between suppression and life satisfaction in the German sample. This finding is in line with the respective cultural values for the experience and expression of emotions. Since Western cultures value self-expression and authenticity (Markus and Kitayama, 1991; Trommsdorff and Rothbaum, 2008), suppressing emotional expressions may be discouraged in Western cultural contexts. Sharing both negative and positive emotions provides information on internal states and can promote closeness with others (Gross and John, 2003; English and Eldesouky, 2020). In independent cultures, suppression might disrupt social interaction, burden relationships, and consequently, reduce social support. In turn, less social support might predict lower life satisfaction. Our findings are in line with experimental (Butler et al., 2003) and longitudinal studies (English et al., 2012) that demonstrated detrimental effects of suppression on social functioning among US-Americans. While we replicated these findings in the German cultural context, the cultural moderation was rather small compared to studies that, for instance, sampled US-Americans and HKC (Soto et al., 2011). Soto and colleagues report a correlation with life satisfaction of *r* = −0.34 for US-Americans, whereas we found a correlation of *r* = −0.24 for Germans (see Supplementary Material). The deteriorating effects of suppression might be weaker in Germany than in the USA due to an emphasis on slightly different values. Specifically, Germany places more value on harmony than the USA (Schwartz, 2006).

In line with past research from Western and East Asian cultures (Gross and John, 2003; Chang et al., 2010), reappraisal was positively related to life satisfaction and social support among Germans and HKC. Among Japanese, reappraisal was linked to more social support but was unrelated to life satisfaction. Notably, the positive effect of reappraisal on social support was weaker for Japanese as compared to HKC. Instead of modifying the meaning of an emotional situation, Japanese individuals are often urged to acknowledge negative emotions for self-growth. Common expressions like *nomikomu* [swallow] illustrate the emphasis of Japanese culture on the acceptance of negative emotions due to dialectical beliefs that an emotional balance between positive and negative emotions should be preferred (Miyamoto and Ryff, 2011). For example, Morita therapy, which was developed in Japan, focuses on “accepting things *arugamama* [as they are]” (Lebra, 1976, p. 223). The common Japanese exclamation *shouganai* (roughly: there is nothing one can do) further illustrates the tendency to acknowledge reality as it is. Similarly, past research found a higher preference for secondary control among Japanese as compared to Germans (Seginer et al., 1993; Trommsdorff and Essau, 1998), suggesting that interdependent cultures promote the acceptance of distressing situations instead of changing them through reappraisal. Still, the fact that reappraisal was related to more social support among Japanese, even though the effect was small, suggests that reappraisal may serve some social functions by changing emotional evaluations to adapt to social expectations.

As hypothesized, rumination was related to lower life satisfaction across cultures. Rumination was also associated with less social support among Germans and HKC, but not among Japanese. Former is in line with findings from Western cultures showing that ruminators behave in socially obtrusive ways (Nolen-Hoeksema et al., 2008). The non-significant relationship with social support among Japanese suggests that rumination might unfold no negative consequences for social functioning in this cultural context. Rumination may cover some culturally valued and socially adaptive practices due to an emphasis on self-effacement and self-criticism in Japan (Heine et al., 1999). Ruminating on negative experiences might help identify one's shortcomings and encourage self-improvement to match social expectations.

The fact that we observed similar effects among Germans and HKC for reappraisal and rumination, respectively, could be partly explained by the unique history of Hong Kong. As a global financial and economic center, Hong Kong differs from the insular seclusion of Japan. Moreover, living under British sovereignty for many years might have promoted the development of Western or bicultural identities among HKC (Kobayashi et al., 2021), thus creating similarities to individuals from Western cultures (Germany). More research is needed to understand whether Hong Kong differs from Japan on associations of ER due to Western influences or due to other causes for intracultural variations in value orientations within East Asian cultures.

### Limitations and Future Directions

Except for rumination which refers to the regulation of negative emotions specifically, the scales we used did not differentiate between emotion types. Future studies may benefit from distinguishing specific emotions. For instance, Japanese have been shown to prefer experiencing socially engaging over disengaging emotions, whereas the opposite was found for US-Americans (Kitayama et al., 2006). In a comparison of Japanese and Belgian couples, Japanese were shown to suppress negative emotions more frequently than Belgians. Notably, this effect was even stronger for socially disengaging emotions compared to socially engaging emotions (Schouten et al., 2020), highlighting the relevance of addressing specific emotions in future research.

Our mediation analyses are based on cross-sectional data and should be interpreted carefully as it remains unclear whether the assumed sequence of relationships represents causal effects. Some authors argue that mediation should be calculated for longitudinal data only (Maxwell et al., 2011), while others state that mediation models for cross-sectional studies are reasonable if interpreted cautiously (Hayes, 2017). Either way, we fully acknowledge that it is desirable for future cross-cultural studies to test our findings with a longitudinal design (e.g., see d'Arbeloff et al., 2018). Moreover, our study applied self-report questionnaires to measure ER. Instead, behavioral measures might be more valid to assess suppression as self-report scales are prone to biases (Heine et al., 2002). Additionally, experience sampling approaches can offer insights into ER in natural settings and specific situations (e.g., Catterson et al., 2017). Finally, it should be noted that our study was carried out during the COVID-19 pandemic in 2020. The stress caused by the pandemic may have affected participants' emotional experiences and their regulation.

## Conclusion

Our findings add to past research by highlighting cultural differences in associations of ER, life satisfaction, and social support among university students from Germany, Hong Kong, and Japan. We demonstrate the mediating role of social support for relationships between ER strategies and life satisfaction, suggesting that previously reported cultural differences in the link between ER and well-being measures might be largely explained by cultural differences in the social adaptiveness of ER strategies. Specifically, among Germans, suppression was related to less social support which completely mediated suppression's effect on lower life satisfaction. Future studies might extend our findings by using experimental or longitudinal designs to examine the causal effects of ER on social functioning and well-being across cultural contexts.

## Data Availability Statement

The raw data supporting the conclusions of this article is available from the corresponding author on reasonable request.

## Ethics Statement

The studies involving human participants were reviewed and approved by Ethics Committee of the University of Konstanz. The patients/participants provided their written informed consent to participate in this study.

## Author Contributions

FS: conceptualization of study design, data collection in Germany, data analysis and interpretation, and preparation of written manuscript. GT: conceptual input, general supervisory, and review of manuscript. NW: conceptual input, data collection in Hong Kong, and review of manuscript. GN: conceptual input, data collection in Japan, and review of manuscript. All authors contributed to the article and approved the submitted version.

## Funding

Research was funded by two grants from the University of Konstanz (PANDOTA Grant and Doctoral Fund), awarded to FS.

## Conflict of Interest

The authors declare that the research was conducted in the absence of any commercial or financial relationships that could be construed as a potential conflict of interest.

## Publisher's Note

All claims expressed in this article are solely those of the authors and do not necessarily represent those of their affiliated organizations, or those of the publisher, the editors and the reviewers. Any product that may be evaluated in this article, or claim that may be made by its manufacturer, is not guaranteed or endorsed by the publisher.
