# Supplementary material for: Associations Between Emotion Regulation and Life Satisfaction Among University Students From Germany, Hong Kong, and Japan: The Mediating Role of Social Support
Source: Front Psychol. 2021 Oct 18;12:745888. doi: 10.3389/fpsyg.2021.745888 (PMC8558612; doi:10.3389/fpsyg.2021.745888)
Supplement: Supplementary file 1 [file Data_Sheet_1.docx]

***Supplementary Material***

1. **Comments on Deviations from Preregistration**
   1. **Sample size**

Each sample slightly deviates from the preregistered sample goal of 120 as it took time to notice and terminate the data collection after our target sample size had been reached. In the case of the German sample, a vast majority of female and few male participants had taken part in our study at the beginning of data collection. Therefore, we extended the German data collection beyond our pre-determined goal to recruit more male participants, thereby adjusting the gender ratio.

- 1. **Hypotheses**

We intended to focus on suppression as an ER strategy and thus neglected to add mediation hypotheses for reappraisal and rumination in the preregistration. We added these in the final article to provide a more complete examination of ER strategies and the mediating role of social support. Furthermore, we changed the format (but not the content) of our hypotheses by integrating the subhypotheses in the main hypotheses (e.g., H1.1, H1.2, and so on, were integrated into H1). Doing so had the aim of improving the comprehensibility of our hypotheses and making them less wordy. Lastly, the numbers of our hypotheses changed since we did not include empathic suppression in our article due to nonsignificant findings in every analysis (hypothesis 2 in preregistration). We present the respective results below.

- 1. **Exploratory Analyses**

We refrained from including exploratory analyses for interdependent happiness, frequency of negative and positive emotions, harmony seeking, and rejection avoidance in our article. Adding further analyses would have added only little to the article, while unnecessarily increasing its length and making its focus less clear. However, we added some exploratory analyses for interdependent happiness and frequency of negative and positive emotions in the supplementals (see next pages).

1. **Additional measure information and language versions used**
   1. **Emotion Regulation Questionnaire (ERQ; Gross & John, 2003)**

We applied the German (Abler & Kessler, 2009: αs = .74–76) and Japanese (Yoshizu et al., 2013: αs = .77–78) version, respectively. For the Hong Kong sample, the English version was translated to Chinese and checked via backtranslation. Gross and John (2003) reported Cronbach’s alphas between .68 and .82 for both scales in the original English version.

- 1. **Perseverative Thinking Questionnaire (PTQ; Ehring et al., 2011)**

The PTQ was created in German language and showed Cronbach’s alphas between .94. and .95 (Ehring et al., 2011). The items were translated from German into Japanese. The Chinese version was created by translating the English items. The translated version were checked via backtranslation.

- 1. **Multidimensional Scale of Perceived Social Support (MSPSS; Zimet et al., 1988: α = .88)**

We used the German (Greimel et al., 2016: α = .93) and Japanese (Iwasa et al., 2007: α = .91) version of the MSPSS, respectively. For the Hong Kong sample, the English version was translated to Chinese and checked via backtranslation.

- 1. **Satisfaction with Life Scale (SWLS; Diener et al., 1985: α = .87)**

Participants completed the German (Glaesmer et al., 2011: α = .92), Chinese (Shek, 2004: α = .76), Japanese (Oishi, 2009: αs = .61–76) version, respectively.

**Table S1**

Sample items for each scale

| **Scale** | **Sample Item** |
| --- | --- |
| Reappraisal | When I want to feel more *positive emotions*, I *change the way I’m thinking* about the situation. |
| Suppression | I control my emotions by *not expressing them*. |
| Rumination | I think about many problems without solving any of them. |
| Social support | I have friends with whom I can share my joys and sorrows. |
| Life satisfaction | I am satisfied with my life. |

*Note.* Italicized words are also italicized in the original versions of the scales.

**Table S2**

*Partial correlations (controlled for age and gender) for Germans*

| Variable | 1 | 2 | 3 | 4 | 5 |
| --- | --- | --- | --- | --- | --- |
| 1. Suppression | – |  |  |  |  |
| 2. Reappraisal | -.22^**^ | – |  |  |  |
| 3. Rumination | .10 | -.36^**^ | – |  |  |
| 4. Social support | -.23^**^ | .40^**^ | -.24^**^ | – |  |
| 5. Life satisfaction | -.24^**^ | .33^**^ | -.34^**^ | .51^**^ | – |

^*^*p* < .05. ^**^*p* < .01.

**Table S3**

*Partial correlations (controlled for age and gender) for Hong Kong Chinese*

| Variable | 1 | 2 | 3 | 4 | 5 |
| --- | --- | --- | --- | --- | --- |
| 1. Suppression | – |  |  |  |  |
| 2. Reappraisal | .31^**^ | – |  |  |  |
| 3. Rumination | .15 | -.17 | – |  |  |
| 4. Social support | -.09 | .36^**^ | -.40^**^ | – |  |
| 5. Life satisfaction | .04 | .25^**^ | -.45^**^ | .57^**^ | – |

^*^*p* < .05. ^**^*p* < .01.

**Table S4**

*Partial correlations (controlled for age and gender) for Japanese*

| Variable | 1 | 2 | 3 | 4 | 5 |
| --- | --- | --- | --- | --- | --- |
| 1. Suppression | – |  |  |  |  |
| 2. Reappraisal | .29^**^ | – |  |  |  |
| 3. Rumination | .11 | .22^*^ | – |  |  |
| 4. Social support | -.12 | .18^a^ | -.13 | – |  |
| 5. Life satisfaction | -.07 | .13 | -.27^**^ | .38^**^ | – |

^a^ Here, this effect was nonsignificant at *p* = .050, whereas we found a slightly different and significant effect in our moderation analysis (*p* = .049). The small change in *p*-value can be attributed to the different analyses used.

^*^*p* < .05. ^**^*p* < .01.

1. **Additional Findings: Empathic Suppression of Negative Emotions**

Our study included an additional ER strategy called empathic suppression which refers to masking negative emotion out of empathic concern for hurting others (König, 2011). Empathic suppression was assessed using the 4-item scale from the Emotion Regulation Inventory (ERI; König, 2011; 0 = *does never apply*, 4 = *applies always*). Culture neither moderated the link between empathic suppression and life satisfaction (*p* = .613) nor between empathic suppression and social support (*p* = .156). Moreover, empathic suppression was unrelated to life satisfaction (Germans: *p* = .373; HKC: *p* = .789; Japanese: *p* = .630) and social support (Germans: *p* = .493; HKC: *p* = .169; Japanese: *p* = .215) across cultural groups. Consequently, requirements for mediation were not met.

1. **Additional Findings: Interdependent Happiness and Frequency of Negative and Positive Emotions**

We measured three additional well-being constructs: Interdependent happiness, frequency of negative emotions, and frequency of positive emotions. The following measures were applied:

- 1. **Interdependent happiness**

The 9-item interdependent happiness scale (IHS; Hitokoto & Uchida, 2015) was used to measure an individual’s well-being based on interpersonal harmony, ordinariness, and quiescence (1 = *strongly disagree*, 5 = *strongly agree*). The validity of the scale has been demonstrated among samples from Japan, the US, Germany, and South Korea (Hitokoto & Uchida, 2015). Alpha was .87 (G = .79, HK = .89, J = .91).

- 1. **Frequency of negative and positive emotions**

We assessed the frequency of negative and positive emotions during the last four weeks using the Scale of Positive and Negative Experience (SPANE; Diener et al., 2010) with six items measuring negative and positive emotions, respectively (1 = *very rarely or never*, 5 = *very often or always*). Alphas were .82 (G = .79, HK = .84, J = .79) for negative emotions and .90 (G = .89, HK = .92, J = .89) for positive emotions.

- 1. **Results and Discussion**

We ran moderation analyses with age and gender as covariates to examine whether culture moderates associations between ER strategies and outcomes. Interdependent happiness was associated with more reappraisal (Germans: *b* = 0.16, *p* = .002; Hong Kong Chinese: *b* = 0.32, *p* < .001; Japanese: *b* = 0.13, *p* = .022) and less rumination (Germans: *b* = -0.25, *p* = .001; Hong Kong Chinese: *b* = -0.47, *p* < .001; Japanese: *b* = -0.27, *p* < .001) across cultures. Across cultural groups, interdependent happiness was unrelated to expressive suppression (Germans: *p* = .051; Hong Kong Chinese: *p* = .450; Japanese: *p* = .098) and empathic suppression of negative emotions (Germans: *p* = .933; Hong Kong Chinese: *p* = .497; Japanese: *p* = .179).

Expressive suppression was significantly related to a lower frequency of positive emotions among Germans (*b* = -0.13, *p* = .018), but not among Hong Kong Chinese (*p* = .100) or Japanese (*p* = .883). We found no association between expressive suppression and the frequency of negative emotions across cultural groups. Culture significantly moderated the link between empathic suppression and negative emotions, *F*(2, 392) = 4.19, *p* = .016, ΔR² = .021. Interestingly, empathic suppression of negative emotions was related to less negative emotions among Japanese (*b* = -0.20, *p* = .015), but not among Germans (*p* = .718) or Hong Kong Chinese (*p* = .119). Empathic suppression was unrelated to positive emotions across cultural groups (but marginally related to more positive emotions among Japanese: *b* = 0.14, *p* = .085). Reappraisal was related to less negative emotions among Germans (*b* = -0.18, *p* = .001) and Japanese (*b* = -0.12, *p* = .040), but not among Hong Kong Chinese (*p* = .173). Furthermore, reappraisal was associated with more positive emotions among Germans (*b* = 0.24, *p* < .001) and Hong Kong Chinese (*b* = 0.23, *p* = .001), but not among Japanese (*p* = .138). Across samples, rumination was related to more negative (Germans: *b* = 0.42, *p* < .001; Hong Kong Chinese: *b* = 0.57, *p* < .001; Japanese: *b* = 0.20, *p* = .002) and less positive (Germans: *b* = -0.31, *p* < .001; Hong Kong Chinese: *b* = -0.45, *p* < .001; Japanese: *b* = -0.23, *p* < .001) emotions. Moreover, culture significantly moderated the link between rumination and negative emotions, *F*(2, 392) = 6.76, *p* = .001, ΔR² = .025, indicating a significantly weaker effect of rumination on negative emotions among Japanese as compared to Germans and Hong Kong Chinese, respectively.

**References**

Abler, B., & Kessler, H. (2009). Emotion regulation questionnaire – Eine deutschsprachige Fassung des ERQ von Gross und John [Emotion regulation questionnaire – A German version of the ERQ by Gross and John]. *Diagnostica*, *55*(3), 144–152. <https://doi.org/10.1026/0012-1924.55.3.144>

Diener, E., Emmons, R. A., Larsen, R. J., & Griffin, S. (1985). The Satisfaction With Life Scale. *Journal of Personality Assessment*, *49*(1), 71–75. <https://doi.org/10.1207/s15327752jpa4901_13>

Diener, E., Wirtz, D., Tov, W., Kim-Prieto, C., Choi, D., Oishi, S., & Biswas-Diener, R. (2010). New well-being measures: Short scales to assess flourishing and positive and negative feelings. *Social Indicators Research*, *97*(2), 143–156. <https://doi.org/10.1007/s11205-009-9493-y>

Ehring, T., Zetsche, U., Weidacker, K., Wahl, K., Schönfeld, S., & Ehlers, A. (2011). The Perseverative Thinking Questionnaire (PTQ): Validation of a content-independent measure of repetitive negative thinking. *Journal of Behavior Therapy and Experimental Psychiatry*, *42*(2), 225–232. <https://doi.org/10.1016/j.jbtep.2010.12.003>

Glaesmer, H., Grande, G., Braehler, E., & Roth, M. (2011). The German version of the Satisfaction with Life Scale (SWLS): Psychometric properties, validity, and population-based norms. *European Journal of Psychological Assessment*, *27*(2), 127–132. <https://doi.org/10.1027/1015-5759/a000058>

Greimel, E., Kato, Y., Muller-Gartner, M., Salchinger, B., Roth, R., & Freidl, W. (2016). Internal and external resources as determinants of health and quality of life. *PLoS ONE*, *11*(5), e0153232, Article e0153232. <https://doi.org/10.1371/journal.pone.0153232>

Gross, J. J., & John, O. P. (2003). Individual differences in two emotion regulation processes: Implications for affect, relationships, and well-being. *Journal of Personality and Social Psychology*, *85*(2), 348–362. <https://doi.org/10.1037/0022-3514.85.2.348>

Hitokoto, H., & Uchida, Y. (2015). Interdependent happiness: Theoretical importance and measurement validity. *Journal of Happiness Studies*, *16*(1), 211–239. <https://doi.org/10.1007/s10902-014-9505-8>

Iwasa, H., Gondo, Y., Masui, Y., Inagaki, H., Kawai, C., Otsuka, R., Ogawa, M., Takayama, M., Imuta, H., & Suzuki, T. (2007). Nihon-go ban Multidimensional Scale of Perceived Social Support no shinrai-sei narabi ni datousei [Reliability and validity of the Japanese version of the Multidimensional Scale of Perceived Social Support]. *Kousei no Shihyo*, *54*(6), 26–33.

König, D. (2011). *Die Regulation von negativen und positiven Emotionen. Entwicklung des Emotionsregulations-Inventars und Vergleich von Migränikerinnen mit Kontrollpersonen* [The regulation of negative and positive emotions. Development of the Emotion Regulation Inventory] [Unpublished doctoral dissertation]. Department of Psychology, University of Vienna.

Oishi, S. (2009). *Shiawase wo kagaku suru: Shinrigaku kara wakatta koto* [Doing the science of happiness: What we learned from psychology]. Shinyosha.

Shek, D. T. L. (2004). Chinese cultural beliefs about adversity: Its relationship to psychological well-being, school adjustment and problem behaviour in Hong Kong adolescents with and without economic disadvantage. *Childhood*, *11*(1), 63–80. <https://doi.org/10.1177/0907568204040185>

Yoshizu, J., Sekiguchi, R., & Amemiya, T. (2013). Development of a Japanese version of Emotion Regulation Questionnaire. *Japanese Journal of Research on Emotions*, *20*(2), 56–62. <https://doi.org/10.4092/jsre.20.56>

Zimet, G. D., Dahlem, N. W., Zimet, S. G., & Farley, G. K. (1988). The Multidimensional Scale of Perceived Social Support. *Journal of Personality Assessment*, *52*(1), 30–41. <https://doi.org/10.1207/s15327752jpa5201_2>
